# Regulation of Natural Killer Cell Function by STAT3

**DOI:** 10.3389/fimmu.2016.00128

**Published:** 2016-04-11

**Authors:** Nicholas A. Cacalano

**Affiliations:** ^1^Department of Radiation Oncology, David Geffen School of Medicine at UCLA, Los Angeles, CA, USA

**Keywords:** signal transducer and activator of transcription, STAT3 transcription factor, natural killer cells, chemokines, cytokines, cancer immunology

## Abstract

Natural killer (NK) cells, key members of a distinct hematopoietic lineage, innate lymphoid cells, are not only critical effectors that mediate cytotoxicity toward tumor and virally infected cells but also regulate inflammation, antigen presentation, and the adaptive immune response. It has been shown that NK cells can regulate the development and activation of many other components of the immune response, such as dendritic cells, which in turn, modulate the function of NK cells in multiple synergistic feed back loops driven by cell–cell contact, and the secretion of cytokines and chemokines that control effector function and migration of cells to sites of immune activation. The signal transducer and activator of transcription (STAT)-3 is involved in driving almost all of the pathways that control NK cytolytic activity as well as the reciprocal regulatory interactions between NK cells and other components of the immune system. In the context of tumor immunology, NK cells are a first line of defense that eliminates pre-cancerous and transformed cells early in the process of carcinogenesis, through a mechanism of “immune surveillance.” Even after tumors become established, NK cells are critical components of anticancer immunity: dysfunctional NK cells are often found in the peripheral blood of cancer patients, and the lack of NK cells in the tumor microenvironment often correlates to poor prognosis. The pathways and soluble factors activated in tumor-associated NK cells, cancer cells, and regulatory myeloid cells, which determine the outcome of cancer immunity, are all critically regulated by STAT3. Using the tumor microenvironment as a paradigm, we present here an overview of the research that has revealed fundamental mechanisms through which STAT3 regulates all aspects of NK cell biology, including NK development, activation, target cell killing, and fine tuning of the innate and adaptive immune responses.

## Introduction

Natural killer (NK) cells are a critical component of the innate immune response: a first line of defense to protect the host against viral infection and the expansion of cancerous cells. Direct cytolytic activity against infected or transformed cells, on the basis of altered or absent MHC class I molecules (“missing self”), is only one of a number of functions performed by NK cells: they are also critical regulatory cells that interact with and modulate the activity of other components of the immune system, such as dendritic cells (DCs), monocytes/macrophages, endothelial cells, and T lymphocytes. Such regulatory functions are mediated through direct cell–cell interactions as well as *via* the secretion of immunomodulatory cytokines, which can edit and shape the repertoire of antigen-presenting cells (APCs) and impact the balance of T cell subsets during an adaptive immune response. As a result of this myriad of interactions, NK cells are key regulators of the inflammatory response and have emerged as important members of the innate lymphoid cell (ILC) family, unique lineages of immunomodulatory cells that develop from a distinct compartment within the common lymphoid progenitor population (1, 2).

Evasion of the immune system is one of the classic hallmarks of cancer (3, 4). Tumor cells rapidly evolve to become moving targets by modulating the expression of immunogenic proteins on their surfaces and by producing a host of soluble factors that repress both innate and adaptive immune responses. The critical role played by host defenses in tumor rejection is underscored by studies in both murine disease and gene knockout models of immune function as well as findings in human cancer patients. Specifically, the role of NK cells in early detection (immune surveillance) and elimination of cancerous cells has been demonstrated in many animal models, in which selective deletion of NK cells leads to the spontaneous development of cancer or failure to reject implanted tumor cells (5–8). Likewise, NK cells isolated from human cancer patients often display grossly defective surface marker profile, cytolytic activity, and cytokine production (9–19). Clinically, the critical role of antitumor immunity has been validated by marked advances in cancer therapy, which employ antibodies that target inhibitory immune checkpoints *via* the CD28–CTLA-4 and PD-1–PD-L1 ligand receptor systems. These novel therapies potentiate antitumor immunity mediated through CD8^+^ T cells as well as NK cells and have resulted in remarkably effective, durable antitumor immune responses (20–26). Like immune checkpoint inhibitors, therapeutics that target kinases and transcription factors also show great promise as cancer treatments by targeting both the tumor cells as well as components of host immunity. Mechanistically, the molecular basis for NK cell dysfunction in cancer patients is a highly complex phenomenon that integrates both direct effects on the NK cells as well as a range of cell–cell interactions and soluble factors that regulate NK activity. NK cells have become an attractive target for immunotherapy strategies as they are known to mediate direct tumor killing as well as exert a critical “helper” function for adaptive immune responses (27–30).

Unfortunately, therapeutic efforts to potentiate NK-mediated killing of tumor cells have met with little success. Several approaches, involving both *in vivo* and *ex vivo* methods to stimulate antitumor NK activity have been disappointing, largely due to (1) molecular evolution of tumors to promote an immunosuppressive microenvironment and (2) the complexity of NK biology and its multiple functions in both innate and adaptive immunity. NK cells are not just tumoricidal lytic machines, and their profound effects on targeting virus-infected cells, cancer stem cells, cytokine and chemokine regulation, and the differentiation of normal and cancerous tissue are only now being fully appreciated.

Specific subpopulations of NK cells, generally distinguished by a CD56bright/CD16^−^ surface profile secrete critical cytokines that can promote the differentiation of normal and transformed tissues, which also impacts antitumor immunity and cancer progression (31). This small sublineage of NK cells can be found in the periphery and represents about 10% of the peripheral NK cells, but they can also be induced following the interaction with sensitive target cells in a process known as “split anergy” (32–38). Thus, NK cells are not only cytotoxic cancer killers but also drive activation and maturation of DCs and T cells, and can induce the differentiation of normal and cancerous stem cells through the secretion of cytokines such as IFN-γ. The finding that cancer stem cells are resistant to chemotherapy and radiation, but are highly sensitive to NK-mediated cytolysis, has suggested that NK cells can be manipulated to target this key population of cells *in vivo*, either by direct NK-mediated killing or by cytokine-driven differentiation to a population that no longer has self-renewal capacity. A key to reprograming NK cells to effectively kill tumors or induce the differentiation of cancer stem cells, however, critically depends upon our understanding at the molecular level how cancers and the tumor microenvironment repress host anticancer immunity.

Here, we review current advances in the understanding of how the ubiquitously expressed transcription factor signal transducer and activator of transcription (STAT)-3 regulates NK cell biology at multiple levels, including activation of cytolytic function, cytokine-mediated effects, and interactions with other components of the immune system. STAT3 is known to limit all aspects of the NK response, but in this review, we focus on the effects of STAT3 in the context of immune surveillance of cancer and modulation of NK activity in the tumor microenvironment.

Signal transducer and activator of transcription-3 is a downstream effector of multiple cytokines and growth factors that modulate NK cell activity and also drives tumorigenesis and resistance of cancer cells to therapy. It is becoming clear that any efforts to block STAT3 activity in cancer cells are likely to have synergistic and/or deleterious off-target effects on host immune responses mediated through NK cells. It is thus critical to understand the effects of STAT3 signaling on NK activity in order to apply STAT3-targeted therapies in the most effective way.

## Activation of STAT3: The Jak/STAT Pathway

Signal transducer and activator of transcription-3 is part of a family of cytokine and growth factor-activated transcription factors, which includes STAT1, STAT2, STAT4, STAT5a/b, and STAT6, which are signaling proteins, targets of phosphorylation by Jak and receptor tyrosine kinases (RTKs), as well as transcription factors whose effects on gene expression profoundly impact the immune response, the outcome of viral infections, embryonic development, and cancer biology (39–43). STAT3 is activated by a wide range of cytokines, in particular those that recognize gp130, such as IL-6, IL-11, oncostatin M (OSM), leukemia inhibitory factor (LIF), and ciliary neurotrophic factor (CNTF), as well as cytokines that engage the common gamma (γc) chain, including IL-2, IL-4, IL-7, IL-9, and IL-15. STAT3 is also activated by RTKs, such as the epidermal growth factor receptor (EGFR) and VEGFR, cytoplasmic kinases, such as Src, as well as some cancer therapies, such as ionizing radiation (IR). STAT3 signaling is controlled by several endogenous feedback inhibitors, such as the suppressors of cytokine signaling (SOCS), protein inhibitors of activated STATs (PIAS), and phosphatases such as SHP-2, which dephosphorylates and inactivates cytokine receptor chains (39–43).

In the context of tumor biology, STAT3 is often constitutively activated and phosphorylated in a wide range of epithelial and hematopoietic cancers, largely *via* paracrine or autocrine stimulation by STAT3-activating cytokines and growth factors. STAT3 drives tumor cell survival and proliferation, epithelial-to-mesenchymal transition (EMT), metastasis, and resistance to chemotherapy and radiation (44–54). In recent years, previously unappreciated critical functions of STAT3 in anticancer immune responses have been revealed in both lymphoid and myeloid lineages. Thus, STAT3-targeted cancer therapies may have a dual function: direct cytostatic or cytotoxic effects on tumor cells and stimulation of cancer destruction and clearance by the immune system. However, the role of STAT3 in anticancer immunity is complex and involves multiple cell lineages, including NK cells. STAT3 targeting in tumors may therefore also have unanticipated, deleterious effects on tumor immunity. Below, we discuss the impact of STAT3 signaling on NK activation and function in the tumor microenvironment.

## Negative Regulation of NK Cell Function by STAT3/Intrinsic Factors

Several studies have shown that tumor-infiltrating lymphocytes and myeloid cells display constitutive STAT3 phosphorylation, including T cells, DCs, neutrophils, macrophages, and NK cells, which is often driven by the production of cytokines and growth factors by both tumor cells and the infiltrating cells themselves, such as IL-6, IL-10, VEGF, and HGF, among others (55). STAT3 can induce an immunosuppressive tumor microenvironment with both direct and indirect effects on NK cells.

To address the role of STAT3 activation in the hematopoietic compartment, Kortylewski et al. used an inducible system to delete the STAT3 gene in bone marrow-derived cells (55–58). Mice expressing a floxed STAT3 gene in combination with Cre driven from the interferon-inducible Mx1 promoter were treated with poly I:C to induce interferon and activate STAT3 gene deletion in hematopoietic cells. Targeting STAT3 in the bone marrow did not have any effect on NK cell development *in vivo*; overall numbers and cytotoxic activity toward standard YAC-1 lymphoma targets in the knockout mice were comparable to WT animals. However, following tumor challenge with the B16 melanoma cell line, splenic NK cells from STAT3-targeted mice displayed enhanced cytolytic activity compared to WT tumor-bearing mice. Further, subcutaneous growth of B16 melanoma cells or the MB49 bladder cancer cell line was virtually abolished in STAT3-targeted mice, suggesting that STAT3 exerts a cell-autonomous immunosuppressive effect in hematopoietic cells in the context of an antitumor response.

To exclude the possibility that tumor rejection in STAT3^−/−^ mice was due to off-target effects of STAT3 deletion on non-hematopoietic tissues, tumor growth studies in lethally irradiated WT mice reconstituted with either STAT3^+/+^ or STAT3^−/−^ bone marrow cells were performed, and it was found that growth of B16 or MB49 tumors was completely blocked in mice reconstituted with STAT3^−/−^ bone marrow cells. Although antibody depletion experiments showed that T cells played a critical role in tumor rejection, NK cells were also involved, as NK depletion using anti-asialo-GM1 antibodies partially abrogated the effects of a STAT3 small molecule inhibitor (CPA-7) on rejection of MB49 tumors.

In order to determine the contribution of STAT3 to NK-specific antitumor responses, Gotthardt et al. generated a targeted STAT3 deletion specifically in the NK compartment, which resulted in increased immunosurveillance in murine melanoma and leukemia models (59). In this study, genetic ablation of STAT3 was accomplished using STAT3fl/fl mice crossed to mice expressing Cre driven from the NKp46 natural cytotoxicity receptor (NCR)-1 promoter, which deleted STAT3 exclusively in NK cells (STAT3fl/fl × Ncr1-iCreTg), sparing other hematopoietic lineages. In these mice, NK cell development, proliferation, and numbers were normal; NK cells at all stages of development are present in numbers similar to WT mice, and expression of key NK developmental and functional markers, such as NK1.1, NKp46, CD27, CD11b, NKG2D, as well as inhibitory receptors, such as LY49A, C, G2, and I, KLRG1 and NKG2A, were identical to NK cells from STAT3^+/+^ animals as well as STAT3fl/fl–Mx1-Cre mice. Some differences were noted, however, as the numbers of DNAM-1(CD226)^+^ NK cells were increased in STAT3-deficient mice compared to WT. This finding is intriguing, as DNAM-1 is a cell surface receptor that mediates NK cell cytolytic activity by engaging the CD155 (PVR) and CD112 (Nectin-2) ligands on target cells (60–65). In addition, the levels of two key mediators of NK-dependent cytotoxicity, granzyme B, and perforin were elevated at the protein level in STAT3-deficient NK cells, supporting the idea that STAT3 negatively regulates NK activity. In the B16F10 melanoma model, the number and size of metastatic lung nodules was greatly reduced in mice with STAT3-deficient NK cells, following intravenous injection of tumor cells. This was accompanied by an increase in the percentage of DNAM-1^+^ circulating NK cells in the STAT3-targeted mice and enhanced *in vitro* cytotoxicity toward B16F10 tumor targets. B16F10 cells express high levels of the DNAM-1 ligand CD155 and thus enhanced lysis of B16F10 tumor cells by STAT3^−/−^ NK cells was abrogated by treatment with a DNAM-1 blocking antibody. It is of note that the authors found no evidence for enhanced *in vitro* cytotoxicity of STAT3-deficient NK cells against other targets, such as YAC-1 and Rma cells, which express low levels of CD155, suggesting that blocking STAT3 activity in NK cells may only increase DNAM-1/CD155-dependent killing. STAT3-deficient NK cells were also more efficient at killing leukemic cells *in vivo*. The size of subcutaneous tumors following injection of v-abl-transformed cells was greatly reduced in mice with the NK-specific STAT3 knockout. Consistent with the data obtained with subcutaneous tumors, it was shown that targeting STAT3 also prolonged the survival of mice injected intravenously with v-abl-transformed cells or infected with a v-abl-encoding retrovirus.

## STAT3-Dependent Extrinsic Factors That Regulate NK Responses in Cancer

Intrinsically, STAT3 activation in NK cells often appears to have a negative regulatory role, as deletion of STAT3 in the NK compartment generally results in improved antitumor immunity. However, STAT3 activation in tumor cells, as well as in other components of the immune response, can provide feedback regulation of NK cells in an indirect manner as well. The antitumor activity and cytokine profile of NK cells depends on a complex interplay among activating and inhibitory cell surface receptors as well as a cocktail of soluble factors. The integration of multiple signals regulates the migration of NK cells to the tumor microenvironment, NK-mediated recognition of target cells, and downstream signaling that controls lytic and helper functions. STAT3 has been shown to play a role in all of these processes.

## Effects of STAT3 Activation in Tumor Cells

Tumor cells often constitutively produce survival or growth factors that enhance tumorigenesis, resistance to therapy, EMT, and metastasis (47, 57, 58, 66). Activated STAT3 in tumor cells impacts anticancer immunity as well, due to its ability to regulate cytokine and chemokine expression by the tumor as well as the expression of growth factors and surface markers on infiltrating immune cells. It has been known for some time that constitutive STAT3 activation in tumor cells can regulate their susceptibility to NK-mediated cytolysis and the infiltration and migration of NK cells within the tumor microenvironment. Constitutive STAT3 phosphorylation in tumor cells is usually driven by the overproduction of key cytokines and growth factors, such as IL-6, IL-10, EGF, HGF, Her2/Neu, and VEGF, or their receptors, as well as aberrantly activated cytoplasmic kinases, such as Src, among others (57). STAT3 activation in tumor cells can regulate their sensitivity to NK-mediated killing by altering the expression of NK-activating ligands on the cell surface. In addition, STAT3-driven cytokine expression in tumors induces the secretion of immunosuppressive cytokines and represses proinflammatory cytokine expression, leading toward a dampening of immune responses in the tumor microenvironment. The relationship between STAT3 activation in tumor cells and components of the innate and adaptive immune responses is complex and multifactorial.

## Effects of STAT3 Activation on Migration of NK Cells to the Tumor Microenvironment

A key proximal event in antitumor immunity is the migration of lymphoid and myeloid cells to the tumor microenvironment. NK cells depend on chemotactic factors secreted by tumor cells, stroma, and other lymphoid and myeloid cells to reach the site of action. While proinflammatory signaling promotes the migration of NK cells to the microenvironment, continued proinflammatory “costimulatory” signals trigger NK-mediated cytokine production and lytic function. Major factors that control NK cell migration to the tumor microenvironment are chemokines and cytokines. NK cells express a range of receptors for soluble factors and thus can respond to a number of chemotactic factors, such as TNF-α, IL-12, RANTES, macrophage chemotactic protein (MCP)2–3, IL-2, MIP1alpha, IL-8, CXCL9, CXCL12, CCL2 (MCP-1), CCL3, CX3CL1 (fractalkine), CXCL10, CXCL12, CCL21 (SLC), and CCL19 (ELC) (67–71). Dysregulated NK migration to the tumor microenvironment has been suggested by studies that found low numbers of NK cells associated with tumors in colorectal cancer (72). Further, experimental models of cancer have shown that gene therapy with CCL2 and CX3CL1/fractalkine can stimulate tumor rejection by increased infiltration and activation of NK cells (73–76).

Burdelya et al. demonstrated that several NK chemotactic factors secreted by tumor cells are regulated by STAT3 in the B16 and K1735 murine melanoma models (77). It was shown that increased STAT3 activity of K1735 subclones correlated to the decreased lymphocyte infiltration of subcutaneous tumors. Treatment of tumor cell lines with a platinum-containing STAT3 inhibitor (CPA-7) or expression of dominant-negative STAT3 (STAT3β) splice variant resulted in increased secretion of chemoattractants, such as RANTES, IFN-γ-inducible protein (IP-10), MCP-1, MIP-2, and T cell activation (TCA)-3. *In vivo* alteration of the cytokine and chemokine profile induced migration of T cells, NK cells, neutrophils, and macrophages to the tumor microenvironment.

Similarly, Ihara et al. demonstrated similar effects of STAT3 targeting in a carcinogen-driven model of non-small cell lung carcinoma (NSCLC) (78). Using a lung-specific, tetracycline-inducible Cre-lox system to delete STAT3, it was found that the growth of urethane-induced tumors in the lungs of STAT3-targeted mice was significantly reduced relative to tumors generated in WT mice. Cell autonomous and non-immune effects of STAT3 deletion in tumor cells were excluded, as markers of proliferation, angiogenesis, and apoptosis were similar in tumors from WT or STAT3^−/−^ mice. In contrast, numbers of lymphoid and myeloid cells in bronchoalveolar lavage fluid were increased in the STAT3^−/−^ mice, as were the levels of proinflammatory cytokines such as IFN-γ, TNF-α, and IL-6. Microarray analysis of the tumors revealed that targeting STAT3 increased the expression of multiple chemokines, such as CCL2, CCL9, CCL12, CCL17, and CCL21c, and induced downregulation of MHC class I molecules H2-D1 and H2-K1 in the tumor cells. These results indicated that STAT3 inhibition can increase NK cell migration to the tumor microenvironment and enhance tumor cell sensitivity to NK-mediated lysis. We have recently shown that knock down of key proteins in tumor cells and non-transformed healthy cells drives dedifferentiation of tumors and results in the activation of NK cell function (37).

On the other hand, some studies have shown that STAT3 can enhance NK-mediated immunosurveillance, specifically in a model of BCR/Abl-dependent leukemia. Putz et al. measured subcutaneous tumor formation in bone marrow cells transformed by BCR/Abl^p185^ or v-Abl^p160^ (79). STAT3 was targeted by interferon treatment of STAT3fl/fl–Mx1-Cre bone marrow cells or by transfecting with a STAT3-specific shRNA lentiviral construct. STAT3-targeted tumors grew larger than WT tumors, with a more invasive phenotype and a higher mitotic index. Cytokine profiling revealed lower levels of proinflammatory mediators IL-6, IFN-γ, IL-17, and CCL5 produced by STAT3-deficient tumors relative to WT controls. Numbers of infiltrating NK cells, as well as CD4^+^ and CD8^+^ T cells, were significantly reduced in the tumor microenvironment, with no change in the numbers of Gr1^+^/CD11b^+^ MDSCs. STAT3-targeted tumor cells were more resistant to lysis by purified primary NK cells *in vitro*, and the growth differences between STAT3-targeted and WT tumors *in vivo* were abrogated in mice lacking NK cells. This study supports previous work implicating NK cells as the major lymphoid population responsible for eradication of BCR/Abl-driven leukemias and suggests that STAT3-targeting strategies may severely impair NK-mediated antitumor immune responses in BCR/Abl leukemias. These conflicting findings indicate that STAT3-targeting strategies in cancer can result in either enhancement or inhibition of anticancer immunity, depending on context and tumor type.

## Regulation of NK Recognition Molecules and Immune Checkpoint Receptors by STAT3

### NKG2D/MICA

A critical mechanism of NK-mediated recognition of target cells is *via* the NKG2D NK-activating receptor (80–90). NKG2D is a transmembrane receptor expressed on the surface of NK cells, which physically associates with the DAP10 signaling chain in humans. The fully functional complex is a hexamer, with one NKG2D homodimer physically associated with two Dap10 homodimers. Recognition of NKG2D ligands, which include the MHC class I polypeptide-related chain (MIC)A, MICB, and members of the Rae-1, ULBP, Mult1, and H60 families, induces phosphorylation of a Y–X–X–M motif on the cytoplasmic tail of DAP10, and activates NK degranulation and killing of target cells. NKG2D engagement triggers both PI-3 kinase and MAPK signaling pathways and can override inhibitory signals *via* MHC class I recognition by killer inhibitory receptors (KIRs). The importance of NKG2D-mediated elimination of tumor cells is underscored by findings in NKG2D gene-targeted mice. In animals lacking NKG2D receptors, NK cell development is normal, but immune surveillance of epithelial and lymphoid cancers is defective (91).

The MICA NKG2D ligand is a stress-induced cell surface molecule that is upregulated in response to genotoxic stimuli, such as chemotherapy or radiation, as well as chromatin disruption and heat shock (89–91). The importance of MICA/NKG2D engagement in the control of cancer has been demonstrated by several studies showing that increased soluble MICA protein in the serum of cancer patients leads to downregulation of NKG2D on NK cells and correlates to poor prognosis (92). STAT3 has been shown to regulate NK lytic activity *via* transcriptional effects on NK recognition receptors and activating NK ligands on tumor cells.

Bedel et al. demonstrated that pharmacologic or genetic inhibition of STAT3 in a human colorectal cell line (HT29) resulted in increased NK cell degranulation (CD107a expression) and IFN-γ production in an *in vitro* coculture assay (93). HT29 is highly resistant to NK-mediated lysis, which correlates to high levels of constitutive STAT3 phosphorylation and protein expression relative to NK-sensitive human tumors. Targeting STAT3 in HT29 with siRNA increased IFN-γ production of freshly isolated human NK cells when cocultured with HT29. Pretreatment of cocultures with anti-NKG2D antibody inhibited IFN-γ secretion and NK-mediated cytotoxicity of STAT3-targeted HT29. The effect of anti-NKG2D antibodies was attributed to increased expression of NKG2D ligands MICA and ULBP2 in STAT3-deficient HT29 cells; MICA expression was increased approximately ninefold, following treatment HT29 with a pharmacologic inhibitor of STAT3 (STA21). STAT3 inhibition increased MICA expression in human GBM and breast cancer cell lines as well, and STAT3 was shown to bind directly to a putative STAT-binding site in the MICA promoter. Pharmacologic STAT3 inhibition increased MICA promoter activity, and expression of a constitutively active STAT3 mutant (STAT3C) blocked MICA expression in a human stromal cell line, demonstrating that STAT3 acts as a MICA transcriptional repressor in this system.

Likewise, Fionda et al. demonstrated a molecular link between glycogen synthase kinase (GSK)-3, STAT3, and MICA regulation in multiple myeloma (MM) (94). It was found that pharmacologic inhibition of GSK-3 downregulated MICA expression and increased the sensitivity of human MM cell lines to NK-mediated cytotoxicity. The effect of GSK-3 inhibition was dependent on downregulation of STAT3 phosphorylation and reduced STAT3 binding to the MICA promoter. It was also shown that expression of a constitutively active STAT3 mutant repressed MICA expression in MM cell lines and reversed the effect of GSK-3 inhibitors in MM.

In contrast, some STAT3-activating stimuli can increase NKG2D expression on NK cells in both mice and humans. Zhu et al. have shown that IL-10 and IL-21 can induce high levels of NKG2D, DAP10, and DAP12 expression on the surface of primary human NK cells, and small-molecule STAT3 inhibitors downregulated NKG2D expression on primary human NK cells from healthy donors (95). Likewise, STAT3-deficient NK cells from gene-targeted mice displayed lower NKG2D surface expression than WT controls. The *in vivo* relevance of STAT3-mediated NKG2D regulation was supported by the finding that NK cells from patients with inactivating STAT3 mutations in conditions, such as hyper IgE syndrome (HIES), displayed lower NKG2D surface expression relative to normal NKs.

In agreement with these studies, IL-21 was shown to augment NKG2D-dependent killing in several murine tumor models (96). Interleukin-21 pretreatment significantly extended survival of mice in a lymphoma model and blocked tumor metastasis in murine models of breast, lung, and prostate cancer. The protective effects of IL-21 were reversed upon treatment of mice with anti-NKG2D antibodies or NK-depleting anti-NK1.1 antibodies. These studies suggest that IL-21-driven STAT3 activation regulates NKG2D expression, although other IL-21-dependent pathways, such as ERK MAP kinase and PI-3 kinase/Akt, are likely to also play a role.

On the other hand, Burgess et al. have shown that IL-21 can inhibit the expression of NKG2D and the adapter/signaling protein DAP10 on IL-2-activated primary human NK cells (97). Interestingly, IL-21 inhibited DAP10 gene promoter activity and reduced surface expression of NKG2D but increased the NK NCR NKp30 and 2B4, suggesting that IL-21 can reprogram NK specificity by editing the recognition receptor repertoire on the cell surface. The different effects of IL-21 in these studies might be explained by the complex interplay between STAT molecules. Interleukin-21 can activate both STAT1 and STAT3, which exert opposing effects in T cells, in particular on the expression of different STAT-dependent genes such as IFN-γ. Thus, it is possible that whether STAT3 has a positive or negative effect on NKG2D or DAP-10 expression depends on the STAT1/STAT3 balance at the time of stimulation (98).

Taken together, STAT3 targeting of tumors may promote NK-mediated cytotoxicity by increasing MICA expression on target cells but can have both positive and negative effects on NK-mediated recognition of tumors by modulating the expression of NK-activating receptors. These conflicting data indicate that the role of STAT3 in the regulation of NK ligands and receptors likely depends on cellular context, the balance between STAT1 and STAT3 activation, method of stimulation, and assay conditions used in the studies.

## STAT3-Mediated Regulation of the PD-1/PD-L1 Immune Checkpoint

The programmed cell death (PD)-1 (CD279)/PD-L1 (B7-H1 and CD274) immune checkpoint pathway has been the focus of intense investigation and clinical application in the last several years. Inducibly expressed on activated T cells, PD-1 is considered a marker of lymphoid cell “exhaustion” or chronic stimulation. Engagement of PD-1 on T cells *via* its ligands PD-L1 or PD-L2 on the surface of lymphoid cells, macrophages, IFN-stimulated epithelial cells, DCs, or tumor cells blocks T cell receptor (TCR) signaling on cytolytic CD8^+^ cells *via* the recruitment of the SHP-2 phosphatase and dephosphorylation of the TCR complex (99–106). Tumor cells from various tissues upregulate PD-L1 expression, which inhibits T cell-mediated antitumor immunity. Much is known of PD-1 signaling in T cells, and remarkable, durable antitumor immune responses have been achieved in melanoma and lung cancer using therapeutic antibodies that block PD-1/PD-L1 engagement (107–115). PD-1/PD-L1 blockade, as a monotherapy or in combination with IR and epigenetic targeting, has shown great promise as a novel emerging cancer treatment and is currently being tested clinically for several other solid and hematopoietic cancers (116–119). Although PD-1 is known to be upregulated on NK cells, its role in the regulation of NK-mediated immune surveillance and tumor killing is poorly understood.

At the molecular level, novel high-resolution, high-throughput imaging studies have demonstrated that PD-1 on the surface of NK cells is situated within the immunologic synapse upon engagement with a target cell. This work provides evidence that PD-1 is physically associated with the cytolytic signaling machinery, with access to NK activating receptors and provides evidence that the composition of the NK immunologic synapse can promote PD-1-mediated dephosphorylation of NK recognition structures and blunt lysis of tumor cells (120).

**Figure 1:**
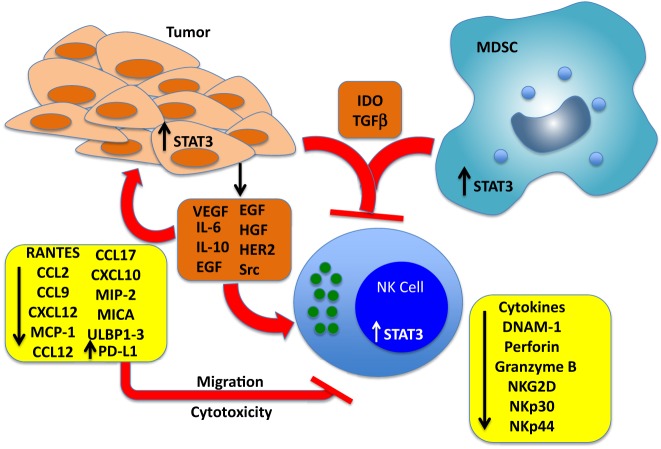
**Effects of cancer cell-derived factors that drive STAT3 activation in tumors, NK cells, and myeloid-derived suppressor cells (MDSCs)**. Tumors produce multiple soluble factors that activate STAT3 in cells of the tumor microenvironment. Among the direct effects of STAT3 activation in NK cells include blunting the expression of key NK activating receptors DNAM-1 NKp30, NKp44, and NKG2D, and inhibition of perforin A and granzyme B expression, which blunts cytotoxic activity. STAT3 activation in tumor cells represses the expression of NK chemotactic factors, which reduces the recruitment of NK cells to the tumor microenvironment, and blunts the expression of NKG2D ligands MICA and ULBP1/2/3, which renders the cells resistant to NK-mediated killing. STAT3 has also been shown to increase the levels of PD-L1, which can engage PD-1 expressed on NK cells and inhibit cytotoxic responses. Finally, IDO and TGF-beta produced by both tumor cells and MDSCs block NK cell development, proliferation, and activation.

Marzec et al. have demonstrated that NPM/ALK-transformed T cell lymphoma cells escape immune destruction *via* induction of PD-L1. PD-L1 expression was STAT3-dependent and STAT3 bound directly to the PD-L1 promoter, which has four putative STAT-binding sites (121). Further, Benson et al. have shown that NK cells from MM patients express PD-1 at a much higher level than NK cells from normal donors (122). The functional significance of PD-1 expression was demonstrated by increased NK-mediated lysis of autologous MM cells following treatment with an anti-PD-L1-blocking antibody (CT-011). The authors also suggest that the immunomodulatory effects of the therapeutic lenalidomide can, in part, be explained by its ability to downregulate PD-L1 expression on primary MM cells, leading to increased NK-mediated lysis and synergy with CT-011. Thus, in at least some tumor models, NK-mediated immune surveillance can be inhibited by STAT3-dependent increases in PD-L1 expression. It is also of interest to note that most highly differentiated tumors express PD-L1 and are found to be resistant to NK cell-mediated cytotoxicity (37). Figure 1 and Table 1 summarize the direct effects of STAT3 activation on the expression of NK activating receptors as well as inhibition of cytotoxicity and migration by downregulation of NK ligands and chemotactic factors in tumor cells.

**Table 1:** **Summary of STAT3-mediated regulation of NK function**.

Effect of STAT3 on molecular profile	Biological effects	Reference
**Direct effects on NK cells**		
Decreased NKG2D and DAP10 expression	Reduced recognition of target cells through NKG2D ligands, reduced signaling *via* DAP10 engagement, and decreased cytotoxicity	(97)
Decreased expression of DNAM-1(CD226)^+^	Impaired cytotoxicity *via* recognition of DNAM-1 ligands Nectin-2 (CD112) and poliovirus receptor (PVR and CD155) on target cells	(59)
Decreased expression of granzyme B and perforin A	Impaired cytotoxic activity and granzyme B-driven apoptosis of target cells	(59, 193)
Decreased production of IFN-γ and TNF-α	Impaired NK activation, maturation, and cytotoxic activity, reduced DC editing, and impaired NK-induced cell differentiation	(32, 33, 37, 93)
**Direct effects on tumor cells**		
Decreased expression of NKG2D ligands MICA, ULBP1, ULBP2, and ULBP3	Impaired NKG2-mediated cytolysis *via* recognition of MICA and ULBP1–3	(93, 94, 193)
Increased PD-L1 expression	Impaired NK-mediated cytotoxicity due to engagement of the inhibitory checkpoint molecule PD-1	(121)
Decreased expression of NK chemotactic factors RANTES, IP-10, MCP-1 (CCL2), MIP-2, TCA-3, CCL9, CCL12, and CCL17	Inhibition of NK migration to the tumor microenvironment	(77, 78, 150)
Increased MHC class I expression	Inhibition of NK-mediated cytotoxicity due to engagement of killer inhibitory receptors (KIRs) by MHC class I molecules	(78)
**Effects on cell–cell communication**		
Increased production of IL-23 by macrophages	Inhibition of IL-12-induced IFN-γ production by NK cells, inhibition of IFN-γ-induced NK maturation and activation, and inhibition of antiviral activity	(151)
IDO expression by tumor cells and MDSCs	Reduced expression of cytotoxicity receptors DNAM-1 NKp30, NKp44, and NKG2D on NK cells; inhibition of NK-mediated cytotoxicity	(168–171)
Decreased expression of CD40, MHC class II, and IL-12 by DCs	Inhibition of NK cell proliferation, activation, and IFN-γ production	(150)
TGF-β production by tumor cells and MDSCs	Inhibition of NK-mediated IFN-γ production	(183–190)
Inhibition of RANTES expression by macrophages	Inhibition of NK migration	(150)

## Effects of STAT3 Activation on Cell–Cell Communication within the Tumor Microenvironment

Perhaps the most important (and complex) role for STAT3 in NK function is as a regulator of cell–cell communication within the tumor microenvironment. STAT3 controls numerous indirect effects on NKs through cytokine secretion by tumor cells, developmental regulation of DCs, as well as chemokine and cytokine production by DCs, macrophages, and MDSCs, all of which exert feedback controls on NK cell migration, activation, and lytic activity (4, 123–129).

**Figure 2:**
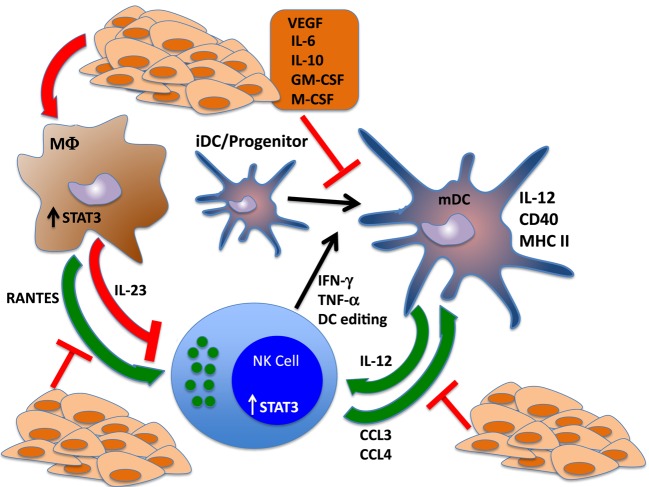
**STAT3 regulates reciprocal interactions between NK cells and other components of the immune response**. NK cells interact with dendritic cells (DCs) in regulatory loops that amplify the activation and function of each cell type. NK cells produce cytokines that drive the differentiation and polarization of DCs to mature, fully functional antigen-presenting cells. Further, NK cells produce potent chemotactic factors CCL3 and CCL4, which recruit DCs to the tumor microenvironment and can shape the DC repertoire *via* cytotoxic elimination of tolerogenic, immature DCs. These functions drive the adaptive immune response by potentiating DC-mediated activation of T cells. Reciprocally, DCs drive NK cell maturation through the production of IL-12. Tumor cell production of cytokines that trigger STAT3 in NK cells and DCs send the antitumor immune response into a tailspin by blocking NK-mediated DC editing and polarization, which inhibits IL-12 release and further impairs NK cell activation and function. STAT3 activation of tumor-associated macrophages also inhibits the immune response by inducing the production of the immunosuppressive cytokine IL-23 and blocking the secretion of NK chemotactic factor RANTES.

Mutually activating, “helper” interactions between NK cells and DCs have been shown to promote the functional capabilities of both cell types as well as inducing the differentiation of CD4^+^ and CD8^+^ T cells, while downregulating immunosuppressive lineages such as Tregs and myeloid-derived suppressor cells (MDSCs) (124, 130–132). Interleukin-18, produced by epithelial cells and macrophages, “primes” NKs responses to DC-derived IL-12 and activates IFN-γ production. Reciprocally, activated NKs produce potent chemoattractants for immature DCs, CCL3, and CCL4, which induce DC migration to the tumor site and drive DC maturation. Interferon gamma and TNF-α produced by activated NK cells can induce polarization of DCs, and NKs also target and kill tolerogenic, immature autologous DCs, increasing the ratio of mature:immature DCs. Thus, NK cells can not only provide helper function to DCs but can also help shape the DC repertoire toward an immunogenic phenotype through a mechanism known as “dendritic cell editing” (28, 125, 133, 134). Inhibition of NK cell function *via* STAT3 activation can therefore skew the DC lineage toward an immature, immunosuppressive phenotype. Likewise, disruption of DC maturation or function can impair NK cell-mediated killing and cytokine production. On the other end of the spectrum, MDSCs can negatively regulate NK cell function through multiple secreted factors, some of which are, at least in part, STAT3 dependent (Figure 2).

## STAT3 Activation in Tumor Cells Regulates DC Function and Maturation

Several studies have shown that STAT3 activation can disrupt interactions between immunocompetent cells in the tumor microenvironment and induce an immunosuppressive milieu. Blocking STAT3 activation in murine tumor models alters the cytokine profile of tumor cells and tumor-infiltrating hematopoietic cells, and re-establishes proinflammatory, tumoricidal activity.

Early studies in cancer patients and murine tumor models revealed that DCs from the tumor microenvironment were defective in antigen presentation. In particular, it was shown that DCs grown from bone marrow precursors of tumor-bearing mice were efficient APCs, but mature splenic DCs from the same mice were defective, suggesting that tumors produced soluble factors that inhibited DC development (135–141). Likewise, several groups found reduced numbers of peripheral DCs in patients with cancers of the kidney, skin, and colon. DCs from cancer patients and animal models were defective in antigen presentation function and failed to effectively induce the B7-1 and B7-2 costimulatory molecules (142, 143). Cancer patients were also shown to produce increased numbers of LIN^−^/HLA-DR^−^ immature myeloid cells in the peripheral blood, indicating defective myelopoiesis induced by tumor factors.

Gabrilovich et al. demonstrated that growth factors produced by tumor cells, in particular vascular endothelial growth factor (VEGF), IL-6, IL-10, macrophage colony stimulating factor (M-CSF), and granulocyte–macrophage colony stimulating factor (GM-CSF), induced severe developmental defects in DCs as well as general dysfunction of myelopoiesis (144–147), and later studies showed that these effects can be traced to STAT3 activation in myeloid progenitors. Supernatants from human breast and colon adenocarcinoma cell lines impaired the differentiation of human CD34^+^ hematopoietic stem cells (HSCs) to functional DCs but did not affect the total number of multipotent stem cells or the antigen presentation function of mature DCs from the periphery, demonstrating a highly specific effect of secreted factors on DC differentiation. The inhibitory effect of the tumor supernatants was reversed by preincubation with anti-VEGF neutralizing antibodies and partially overcome with blocking antibodies specific for M-CSF.

Nefedova et al. subsequently demonstrated that conditioned medium from the murine CT26 colon carcinoma or C3 sarcoma cell lines could trigger Jak2/STAT3 signaling in hematopoietic progenitor cells (HPCs) and resulted in the abnormal accumulation of Gr1^+^/CD11b^+^ immature myeloid cells in an *in vitro* DC differentiation assay (148, 149). Likewise, CD11c^+^/IAd^+^ immature DCs, differentiated in the presence of tumor supernatants, displayed much higher STAT3 DNA binding activity compared to control DCs and were impaired in their ability to stimulate T cell proliferation in an allogeneic mixed lymphocyte reaction (MLR). Expression of a dominant-negative STAT3 mutant in HSCs reversed the effects of tumor supernatants. Conversely, expression of a constitutively active STAT3 mutant resulted in the accumulation of Gr-1^+^/CD11b^+^ immature myeloid cells that poorly differentiated into DCs *in vitro*, demonstrating the causative role of STAT3 in defective DC development.

Wang et al. have shown that in three murine cancer models with constitutive STAT3 activation, B16 (melanoma), SCK-1 9 (breast carcinoma), and CT26 (colon carcinoma), expression of a dominant-negative STAT3 variant, STAT3β, or a STAT3-targeting siRNA increased the levels of the proinflammatory cytokines IFNβ, TNF-α, and IL-6, as well as the chemokines RANTES and CXCL10 (IP-10) in the tumor cells, with no effect on IL-4 and IL-10 production (150). Furthermore, conditioned media from STAT3β-expressing B16 tumor cells induced proinflammatory mediators nitric oxide (NO) and RANTES in peritoneal macrophages and TNF-α in neutrophils. STAT3-targeted B16 tumors *in vivo* also contained higher levels of infiltrating macrophages, neutrophils, and lymphocytes compared to tumors derived from the B16 parental cell line.

Conditioned medium from STAT3-targeted B16 melanoma and CT26 colon carcinoma cells also altered the surface phenotype of DCs, inducing higher expression levels of DC activation and maturation markers IL-12, CD40, and MHC class II molecules. *In vivo*, STAT3β^+^ B16 and CT26 tumor cells triggered DC activation and enhanced infiltration of tumor-specific CD8^+^ T cells. In reciprocal experiments, constitutive STAT3 activation in transformed cells induced the production of secreted factors that inhibited DC maturation, reduced the numbers of CD11c^+^, MHC class II^+^ cells, and blocked IL-12 production in an *in vitro* DC differentiation assay.

The findings from these studies indicate that STAT3 activation is at the apex of a cascade, beginning in tumor cells, which determines the cytokine profile of the cancer and triggers further STAT3 activation in lymphoid and myeloid populations within the tumor microenvironment, promoting immunosuppression and tumor escape. STAT3-driven inhibitory effects on the production of chemokines and inflammatory mediators by tumor cells, inhibition of DC maturation, and the production of IL-12 can block NK activity within the tumor microenvironment (Figure 2).

## STAT3 Determines the Relative Abundance of IL-12 and IL-23 in the Tumor Microenvironment

Signal transducer and activator of transcription-3 signaling has been shown to regulate the cytokine balance and functional interactions between NKs and other hematopoietic cells in the tumor microenvironment. In particular, STAT3 regulates the levels of two key cytokines, IL-12 and the procarcinogenic IL-23, which are related cytokines that share a common ligand chain (IL12p40), as well as a common receptor chain (IL-12Rb1), but exert opposing effects on antitumor immunity (151). Both cytokines are produced primarily by macrophages and DCs, but they differ critically in their signaling properties. Interleukin-12 is a STAT4 activator, while IL-23 is a potent STAT3 inducer. There is also evidence that IL-12 and IL-23 cross inhibit each other during an inflammatory response (152, 153). Interleukin-12 is a critical cytokine that induces the maturation and functional activation of NK cells; it has been shown that NK cell differentiation and effector function are defective in IL-12- and IL-12 receptor-deficient mice, and patients with IL-12/IL-12R mutations are immunodeficient (154–158). In the context of an antitumor immune response, administration of recombinant IL-12 has antitumor effects, and IL-12 knockout mice were found to be more susceptible to ultraviolet light (UV)-induced tumors and skin papillomas (159). Conversely, Langowski et al. have shown that mice with a targeted deletion of the IL-23p19 subunit are completely resistant to TPA-induced skin papillomas, demonstrating a tumor promoting function for IL-23 (160, 161).

A recent study has shown that STAT3 signaling controls the IL-12:IL-23 ratio in the tumor microenvironment (151). When subcutaneously grown B16 melanoma tumors were dissociated and tumor-associated myeloid cells purified, it was found that tumor-infiltrating CD11b^+^ c-macrophages secreted high levels of IL-23, whereas tumor-associated DCs (CD11b^+^c^+^) cells produced very little IL-23. Conversely, neither macrophages nor DCs within the B16 tumor microenvironment secreted significant amounts of IL-12. When STAT3 expression was ablated in the hematopoietic compartment using the STAT3fl/fl–Mx1-Cre system, the levels of macrophage-derived IL-23 was greatly reduced and tumor-associated DCs secreted increased amounts of IL-12. STAT3 was shown to be a direct transcriptional activator of the IL-12p19 gene, and IL-23 in the tumor microenvironment inhibited IL-12 production by tumor-associated DCs. STAT3-dependent reciprocal regulation of IL-23 and IL-12 was demonstrated in melanoma (B16), bladder (MB49), and colon (MB38) cancer models and targeting STAT3 in reduced tumor growth *in vivo*.

These studies demonstrate that the ability of the immune system to reject a tumor depends critically on a delicate balance of secreted factors and cell–cell interactions. Altering these conditions through aberrant STAT3 activation can establish an immunosuppressive circuit between tumor cells and infiltrating macrophages, DCs and NK cells, driven by STAT3 activation in all three subpopulations. Immunosuppressive reprograming of the tumor microenvironment results in a negative feedback loop, wherein STAT3 activation in NK cells inhibits direct tumor cell cytolysis and DC maturation by inhibiting NK-dependent IFN-γ and TNF production and blocking the release of antigenic proteins by NK-mediated lysis of tumor targets; STAT3 activation in the myeloid compartment blocks the production of IL-12 by DCs and triggers the secretion of IL-23 by tumor-associated macrophages, which further impairs NK cell activation and skews the DC repertoire toward an immunosuppressive phenotype by preventing NK-mediated lysis of immature, tolerogenic DCs. Crosstalk between NK cells, macrophages, and DCs has further immunosuppressive effects on the adaptive immune response by inhibiting the proper activation of CD4^+^ and CD8^+^ T cells and promoting the infiltration, development, and activation of MDSCs and CD4^+^, FoxP3^+^ regulatory T cells (TRegs). Thus, a STAT3-activating tumor microenvironment, which begins with the production of cytokines and growth factors by tumor cells, triggers a STAT3 cascade in tumor-infiltrating immune cells that has long-range, profound effects on host antitumor immunity that allows the cancer to escape detection and destruction (Figure 2).

## STAT3-Dependent Regulation of Indoleamine 2,3-Dioxygenase in the Tumor Microenvironment

Myeloid-derived suppressor cells from both humans and mice produce indoleamine 2,3-dioxygenase (IDO), which negatively regulates the function of both myeloid and lymphoid lineages within the tumor microenvironment. IDO catalyzes the rapid catabolism of tryptophan to kynurenine, which inhibits proliferation and effector function of T and NK cells (162–167). Expression of IDO in the tumor microenvironment inhibits NK function by downregulation of activating NK receptors DNAM-1, NKp30, NKp44, and NKG2D, inhibition of IFN-γ production, increased IL-4 and IL-13 secretion, and induction of NK cell apoptosis (168–171). Strikingly, IDO is also overexpressed by tumor cells, as well as tumor-associated fibroblasts, and mesenchymal stem cells (MSC); regardless of the source, IDO has been shown to profoundly inhibit NK-mediated cytokine production, expression of natural cytolytic receptors (NCRs), and tumor cell cytolysis.

Yu et al. identified an “immature” subset of MDSCs (CD33^+^CD13^+^CD14^−^CD15^−^), induced from CD33^+^ progenitors upon coculture with the human breast cancer cell line MDA-MB-231, that produced high levels of IDO (172, 173). In this MDSC subpopulation, STAT3 was required for NFκB-inducing kinase (NIK) phosphorylation and activation of the non-canonical NFκB signaling pathway, which upregulated IDO expression by direct activation of the IDO promoter. A Jak/STAT inhibitor, JSI-124 (cucurbitacin I), blocked STAT3 phosphorylation, NFκB activation, and IDO production by MDSCs. In tumor sections from 30 breast cancer patients, the percentage of CD33^+^/pSTAT3^+^ co-expressing cells was 80, suggesting a critical role for this pathway in driving IDO expression in MDSCs associated with primary human breast tumors.

Constitutive, STAT3-dependent IDO expression in drug-resistant tumor cells has been described by Campia et al. (174). Doxorubicin (Dox)-resistant clones of human colon (HT29), lung (A549), and leukemia (K562) cell lines, a murine chemoresistant breast cancer cell line (JC), and human metastatic mesothelioma (HMM) were shown to express high levels of IDO1 and displayed enhanced subcutaneous growth *in vivo* relative to chemosensitive parental cell lines. Enhanced tumor growth was blocked by treatment of mice with an IDO1 inhibitor, 5-Br-Brassinin. Expression of IDO correlated to enhanced Jak1, STAT1 and STAT3 phosphorylation, and the expression of STAT3 targets that induce IDO expression, such as IL-6, IL-1β, IL-4, IL-13, CD40L, and TNF-α. Interestingly, the expression of PIAS3, an endogenous negative feedback inhibitor of STAT3 signaling, was downregulated in drug-resistant A549 cells relative to the parental cell line. These results suggest that constitutive STAT3 signaling and IDO expression in drug-resistant tumors may be enhanced by the inactivation of STAT3-specific feedback controls such as PIAS3.

Other studies have linked STAT3 and the aryl hydrocarbon receptor (AHR) to IDO expression in human NSCLC and ovarian cancer (175). Litzenberger demonstrated that IDO can drive its own production *via* an IDO–AHR–IL-6–STAT3 signaling pathway. IDO metabolizes tryptophan to kynenurine, which binds to the AHR, transactivating target genes such as IL-6. Interleukin-6 activates STAT3, which drives IDO transcription, sustaining the cycle.

Likewise, Chen et al. have demonstrated that IDO is induced in human breast fibroblasts (RMF-EG) following co-incubation with COX-2-overexpressing breast cancer cells (176). It was shown that COX-2-dependent production of PGE2 in human breast cancer cell lines induced IDO, and targeting of the PGE2 receptor EP4 abrogated IDO induction. Two STAT-binding GAS elements were identified in the IDO promoter. STAT3 binding to the IDO GAS elements was increased in fibroblasts when cocultured with breast cancer cells, and siRNA targeting of STAT3 abolished IDO induction by a EP4 agonist.

In summary, there are multiple pathways by which STAT3 indirectly modulates the function of NK cells, either by the production of tumor-derived immunosuppressive factors, such as IDO, or by driving the development of immunosuppressive myeloid populations, such as MDSCs, which secrete IDO, transforming growth factor (TGF)-β, or other soluble factors that block NK development, cytolytic function, or cytokine production (Figure 1; Table 1).

## Regulation of NK Function by STAT3-Induced TGF-β Production

Another key regulatory pathway that influences NK cytolytic and helper functions involves TGF-β. It has been well documented that TGF-β is produced by both tumor cells and tumor-infiltrating myeloid cells (177–182). The biological effects of TGF-β in tumorigenesis have been extensively characterized, and it has been shown to both inhibit and promote tumor activity, depending on the context. Tumor cells often escape the growth inhibitory effects of TGF-β by converting its signaling to promote invasive, metastatic behavior, and can drive EMT. TGF-β regulates the immune system on many levels and modulates the function of most lymphoid and myeloid lineages.

In the tumor microenvironment, high levels of TGF-β are attributed to several mechanisms, including overexpression by tumor cells and cancer-associated fibroblasts (CAFs), as well as induced expression in tumor-infiltrating MDSCs and regulatory T cells (Tregs). Several immunosuppressive TGF-β target genes have been identified, including FoxP3, which drives the development of Tregs (181, 182).

Transforming growth factor-β impairs NK function by reducing the expression levels of NK activating receptors NKG2D and NKp30, blocking the production of IFN-γ and chemokines, and inhibiting NK cell degranulation by impairing release of granzyme A and granzyme B (183–190). More indirectly, TGF-β also impairs DC-driven activation of T and NK cells by reducing the expression of IL-12, MHC class II molecules, and the costimulatory molecules CD80 and CD86 (191).

In several studies, disruption of STAT3 signaling in tumor cells was shown to drive down TGF-β expression and potentiate NK-mediated killing of tumor targets. Sui et al. demonstrated a link between STAT3, TGF-β, and tumor surveillance by NK cells in a model of hepatocellular carcinoma (HCC) (192). Treatment of two murine HCC cell lines, Hepa1–6 and H22, with a STAT3-binding site decoy oligonucleotide impaired tumor growth *in vivo* and prolonged survival of animals with subcutaneous tumors. Numbers of peripheral NK cells (CD3^−^/DX5^+^) and cytotoxic function of liver- or spleen-derived lymphocytes against H22 target cells was increased in mice bearing STAT3-targeted H22 tumors relative to NK cells from mice injected with the parental H22 cell line. Peripheral and tumor-associated NK cells from STAT3-targeted tumor-bearing mice also expressed elevated levels of NK activation markers NKG2D, CD69, and Fas ligand (FasL). Critical involvement of NK cells in tumor rejection was demonstrated by showing that STAT3 deletion impaired H22 growth in nude mice, which lack T cells but have functional NK cells. Antibody-mediated depletion experiments in immunocompetent mice showed that the inhibitory effect of STAT3 targeting was greatly reduced when NK cells were ablated with an anti-asialo-GMP antibody, and adoptive transfer of purified CD3^−^/DX5^+^ NK cells from tumor-bearing mice inhibited H22 tumor growth and extended survival of recipient mice. The role of TGF-β was demonstrated by antibody blocking experiments in which anti-IL-10 plus anti-TGF-β antibodies could reproduce the effects of STAT3 targeting of H22 cells in *in vivo* tumor growth experiments.

Likewise, Sun et al. demonstrated a critical link between STAT3 blockade and inhibition of TGFβ production in HCC cell lines, leading to increased sensitivity to NK-mediated lysis (193). Human HCC cells lines Hep2G, H7402, and PLC/PRF/5 were more sensitive to killing by human PBMCs and NK cell lines NKL or NK-92 when transfected with a STAT3-binding decoy oligonucleotide. A major effect of STAT3 targeting in HCC was increased expression of NKG2D ligands ULBP1, ULBP2, and ULBP3, and enhanced NK-mediated killing of STAT3-targeted HCC cells was partially reversed by treatment with an anti-ULBP3 neutralizing antibody.

Further, supernatants from STAT3-targeted HCC cells enhanced the cytolytic activity of NK cell lines against HCC tumor targets, suggesting that the profile of secreted factors by the tumor cells was altered by STAT3 inhibition. Supernatants from STAT3-targeted tumor cells increased the expression of NK activation markers, such as CD69, NKG2D, Fas ligand, granzyme B, perforin, and IFN-γ. Cytokine profiling identified TGF-β, IL-10, and IL-8 as downregulated in all HCC cell lines following STAT3 targeting, while IFN-γ was consistently upregulated. The importance of TGF-β in regulating NK-mediated tumor killing was demonstrated by increased HCC cell lysis following anti-TGF-β treatment. Conversely, increased NK-mediated tumor cell killing conferred by STAT3 decoy oligonucleotide was reversed by the addition of exogenous TGF-β. The immunotherapeutic potential of blocking TGF-β has been suggested by some recent studies in preclinical models. Alvarez et al. showed that a neutralizing anti-TGF-β antibody could reverse NK inhibition within the tumor microenvironment in the murine Lewis lung carcinoma (LLC) model of NSCLC (194).

## NK Cell Regulation by Endogenous Feedback Inhibitors of STAT3

Tumor cells become addicted to STAT3 signaling *via* the constitutive expression of STAT3-activating growth factors and cytokines or constitutively active mutations of RTKs or cytoplasmic kinases. However, STAT3 dependence is often further driven by inactivation of endogenous negative feedback mechanisms that normally control STAT3 signaling. Among these mechanisms, the SOCS are a large superfamily of STAT inhibitors, some of which bind directly to activated kinases or receptor chains and blunt cytokine and growth factor-driven STAT activation (195–198). STAT3 dependence in tumors is often accompanied by epigenetic silencing of the SOCS1 or SOCS3 genes *via* CpG island hypermethylation and histone deacetylation, which greatly enhances the cell’s sensitivity to STAT3 activation, increasing both the magnitude and duration of a STAT3-dependent stimulus. Selective pressure to repress SOCS3 expression in STAT3-addicted tumors has been shown in cancers of the brain, esophagus, prostate, lung, bone marrow, colon, liver, cervix, and head/neck (199–206), which suggests that SOCS3 inhibition may not only promote tumor cell proliferation and metastasis but also drive tumor cell-mediated immunosuppression.

Modulation of SOCS3 in tumor-infiltrating myeloid cells may also contribute to an immunosuppressive environment that represses NK activity. Yu et al. have recently shown that SOCS3-deficient myeloid cells (SOCS3^MyeKO^) displayed enhanced STAT3 phosphorylation and increased levels of IL-1, IL-6, TNF-α, and iNOS (207). SOCS3^MyeKO^ bone marrow cells preferentially differentiated into MDSC-like cells when cultured in conditioned medium from TRAMP-C1 prostate cancer cells, and TRAMP-C1 tumor growth was accelerated in SOCS3^MyeKO^ compared to WT mice, indicating that SOCS3 can control the extent of MDSC production and may indirectly regulate NK activity in the tumor microenvironment.

In contrast, it has been shown that SOCS3 may drive the generation of immunosuppressive DCs. Mesenchymal stem/stromal cells can induce HSCs to differentiate into CD11b^+^, Interleukin-10-dependent regulatory DCs (208). This novel differentiation pathway was highly dependent on IL-10-driven chromatin reorganization and activation of the SOCS3 locus and could be reversed by siRNA-mediated SOCS3 targeting. This study suggests that SOCS3 can contribute to an immunosuppressive phenotype by skewing the DC developmental program in hematopoietic progenitors.

Analysis of SOCS3 in NK cells suggests that it may have a distinct, direct role in this lineage as well. Braunschweig et al. demonstrated that SOCS3 can regulate NK cytolytic activity, using the NK-92 human NK cell line (209). Small interfering RNA-mediated targeting of SOCS3 resulted in increased STAT3 phosphorylation and NK-mediated killing of tumor targets, which suggests that SOCS3 may be a negative regulator of NK activity and therapeutic targeting of SOCS3 in NK cells may potentiate killing of tumor targets.

Likewise, Xu et al. demonstrated that the chemokine CCL2 can inhibit NK cytolytic function by inducing SOCS3 and partially blocking perforin expression (210). CCL2 impaired the cytolytic activity of freshly isolated CD56^+^ human NK cells toward K562 targets, which coincided with SOCS3 induction and partially repressed perforin expression. Transfection of a SOCS3-specific siRNA reversed both the perforin repression and inhibition of cytolytic activity.

Chemokines, such as CCL2, which are normally critical for NK migration and tumor elimination, can in fact become immunosuppressive in a tumor microenvironment lacking key NK-activating “costimulatory” cytokines. Without each component of the process, CCL2 may repress NK activation through a SOCS3-dependent mechanism.

These findings suggest a dual role for SOCS3 in tumor immunity. On the one hand, when expressed in cancer cells and MDSC precursors, SOCS3 can promote antitumor immunity by blocking the effects of STAT3-inducing, immunosuppressive cytokines. On the other hand, in NK cells and DC precursors, SOCS3 may be a key mediator of STAT3-driven immunosuppression. The *in vivo* relevance of these observations will depend on the generation tissue-specific knockout animals with targeted SOCS3 deletion in DCs and NK cells.

The PIAS is another family of feedback inhibitors of STAT activation that are aberrantly modulated in tumors. It comprised four members: PIAS1, PIAS3, PIASx, and PIASy that can directly bind to STATs and block phosphorylation, DNA binding, and transactivation (211, 212); STAT3 is targeted primarily by the PIAS3 protein. While PIAS proteins can be overexpressed in some cancers (213), PIAS3 expression is repressed in anaplastic lymphoma, glioblastoma, mesothelioma, and NSCLC, which correlates to constitutive STAT3 activation (214–217). Thus, by promoting STAT3 activation, repression of PIAS3 expression in tumor cells can indirectly contribute to NK cell dysfunction by altering the cytokine profile of the cancer. In addition, some studies suggest that inactivation of PIAS3 may be involved in tumor-associated immunosuppression *via* IDO expression in chemoresistant cancers.

These studies suggest an important link between tumor-mediated immunosuppression and other, early events in tumorigenesis, especially epigenetic silencing of potential tumor suppressor genes. The role of SOCS3 as a negative feedback inhibitor of STAT3 signaling often targets it for CpG island methylation in cells that are addicted to STAT3 signaling and, as an additional selective advantage, may induce or enhance the immunosuppressive potential of STAT3-addicted tumors. Moreover, downregulation of SOCS3 in the myeloid compartment can contribute to increased STAT3 phosphorylation, activation of a MDSC developmental pathway, and the secretion of immunosuppressive cytokines.

## Conclusion

The STAT3 transcription factor has been known for many years to drive tumorigenesis, cancer cell survival, proliferation, metastasis, and resistance to anticancer agents. Only relatively recently has its role in the immune system’s response to cancer been fully appreciated. STAT3 regulates antitumor immunity at virtually every level, affecting both lymphoid and myeloid compartments, innate and adaptive immunity, effector cells and APCs, cell migration to the tumor microenvironment, cytolytic and helper function, and the secretion of cytokines and growth factors that modulate immune responses. NK cells are greatly affected by activated STAT3 both intrinsically and extrinsically. Hematopoietic and NK-specific STAT3 genetic ablation have demonstrated that STAT3 is not critical for normal NK development or proliferation. However, STAT3 negatively regulates NK activation and tumor cell killing, as STAT3-deficient NK cells generally exhibit enhanced cytolytic activity and cytokine secretion *in vitro* and *in vivo*. The molecular basis of this regulation is not well understood, although it likely involves STAT3-mediated repression of NK activating receptors, such as DNAM-1, as well as cytolytic mediators, such as perforin and granzyme B.

Aside from the direct effects of STAT3 activation in NK cells, constitutive STAT3 phosphorylation in transformed cells can indirectly impair NK function by regulating the expression of ligands for NK activating receptors as well as important immune checkpoint proteins. For example, tumors can escape NK-mediated recognition and lysis through STAT3-dependent downregulation of the NKG2D ligand MICA. This is likely to impair a critical mechanism to eliminate tumor cells that have been treated with DNA damaging agents such as radiation. Likewise, STAT3-driven PD-L1 expression in tumor cells may dampen antitumor immunity by engaging the PD-1 receptor on the surface of NK cells.

Perhaps more importantly, STAT3 activation in tumor cells triggers a cascade of cytokine and growth factor production that reprograms the tumor microenvironment, skewing it toward immunosuppression. Cytokines produced by tumor cells activate STAT3 in NK cells, DCs, T cells, and macrophages that profoundly affect each population intrinsically and *via* critical crosstalk mechanisms that shut down both innate and adaptive immunity. STAT3 activation in DCs results in impaired IL-12 production, leading to defective NK activation and reduced cytolytic activity toward tumor targets as well as tolerogenic, immature DCs. Reciprocally, poorly activated NK cells fail to produce critical cytokines (IFN-γ and TNF-α) to DCs and T cells, which impairs DC maturation, antigen presentation, and T cell activation. Likewise, STAT3 activation in macrophages alters the balance between IL-12 and IL-23, which enhances carcinogenesis and impairs NK function. This complex web of inhibitory interactions between tumor cells and components of innate and adaptive immunity can send the immune system into a downward spiral in the tumor microenvironment. Finally, STAT3 activation in tumor cells impairs the migration of virtually every component of the immune response to the tumor microenvironment, such that responding cells cannot reach the site of the cancer; targeting STAT3 in tumors alters their cytokine/growth factor profile, resulting in increased migration to microenvironment and potentially enhancing antigen presentation and immune effector functions.

The role of STAT3 in anticancer immunity and therapy becomes even more complex in light of studies suggesting that STAT3 can promote NK-mediated tumor immunity. Some reports have shown that STAT3 can increase the expression of the NK activating receptor NKG2D and promote NK-mediated immunosurveillance in some lymphoma models. Thus, biological targeting of STAT3 in tumor cells may not only have synergistic effects by potentiating the function of NK cells and other components of the immune response but also produce inhibitory off-target effects on NK cells in certain cancers. This raises the possibility that STAT3 inhibitors may work best when combined with one or more of the novel, potent immune checkpoint inhibitors that target PD-1, PD-L1, or CTLA-4, inhibition of immunosuppressive factors, such as IDO, or treatment with chromatin modifiers that release SOCS genes from epigenetic repression in tumor cells. Molecular profiling of tumor tissue, as well as infiltrating lymphoid and myeloid cells, may be of great value to predict the possible immunologic outcome of treating cancer patients with STAT3 inhibitors.

## Author Contributions

NC researched and wrote the article.

## Conflict of Interest Statement

The author declares that the research was conducted in the absence of any commercial or financial relationships that could be construed as a potential conflict of interest.
